# Investigation of prognostic value of polymorphisms within estrogen metabolizing genes in Lithuanian breast cancer patients

**DOI:** 10.1186/s12881-015-0147-4

**Published:** 2015-02-04

**Authors:** Aistė Savukaitytė, Rasa Ugenskienė, Roberta Jankauskaitė, Darius Čereškevičius, Eglė Šepetauskienė, Elona Juozaitytė

**Affiliations:** Oncology Research Laboratory, Oncology Institute, Lithuanian University of Health Sciences, Eiveniu g. 2 LT-50009, Kaunas, Lithuania; Center of Informatics Technologies, Lithuanian University of Health Sciences, Sukileliu pr. 17 LT-50009, Kaunas, Lithuania; Oncology Institute, Lithuanian University of Health Sciences, Eiveniu g. 2 LT-50009, Kaunas, Lithuania

**Keywords:** *GSTM1*, *GSTT1*, *GSTP1*, *SULT1A1*, *UGT1A1*, Estrogen metabolism, Polymorphism, Breast cancer

## Abstract

**Background:**

Breast cancer is the most frequent oncological disease among women. Estrogens are known to play an important role in breast cancer development. Recognition of the relationship between polymorphisms within estrogen metabolizing genes and conventional prognostic factors of breast cancer might improve our knowledge on individualized breast cancer prognosis. Therefore, we aimed to investigate possible associations between germline genetic polymorphisms within *GSTM1, GSTT1*, *GSTP1*, *SULT1A1* and *UGT1A1* genes and breast cancer clinicopathological characteristics together with disease progression.

**Methods:**

Our study involved 80 young (younger than 50 years of age) breast cancer patients. PCR-based Restriction Fragment Length Polymorphism (RFLP) assay was used to determine *GSTP1* and *SULT1A1* genotypes. *GSTM1* and *GSTT1* null genotypes were detected by multiplex PCR. *UGT1A1* polymorphism was investigated with microsatellite analysis. Relationships between genotypes and breast cancer clinicopathological features along with disease progression were estimated by Pearson‘s Chi-square test. Logistic regression analyses were performed to estimate the odds ratios associating different genotypes with clinicopathological characteristics and disease progression.

**Results:**

The study showed individuals with *GSTT1* null genotype to have approximately 3.5 times higher risk for breast cancer progression than those with wild type genotype (OR = 3.472, 95% CI 1.043-11.559, P = 0.043). Moreover, *SULT1A1* G638A AA genotype significantly increased the chances of HER2 molecular subtype breast cancer when compared to GG genotype (OR = 19.971, 95% CI 1.716-232.480, P = 0.017). Heterozygotes for G*STP1* A313G genotype were more likely to have positive lymph nodes in comparison to AA genotype carriers (OR = 2.803, 95% CI 1.049-7.487, P = 0.040). No significant correlation was determined for *UGT1A1* A(TA)nTAA and *GSTM1 +/-* polymorphism alone or combined *GTTT1* null and *GSTM1* null genotype.

**Conclusions:**

Conclusively, our findings suggest that *GSTT1* null genotype and *SULT1A1* G638A AA genotype could be uselful genetic markers for breast cancer prognosis. Further analyses on larger sample size are required to highlight the effect of *GSTP1* G allele on breast cancer prognosis.

**Electronic supplementary material:**

The online version of this article (doi:10.1186/s12881-015-0147-4) contains supplementary material, which is available to authorized users.

## Background

Breast cancer (BC) is the most frequent oncological disease among women. It is widely accepted that prolonged exposure to estrogens and their oxidative metabolites play an important role in BC developement. The carcinogenic effect of estrogens is thought to manifest in part through the ability of intermediate estrogen metabolites to form superoxide radicals and depurinating adducts that damage DNA [1,2]. Genetic polymorphisms in low penetrance genes, involved in estrogen production and metabolite elimination, affect the level of estrogens in breast tissue. Although many studies have shown associations between polymorphisms within these genes and BC risk, only a few have elucidated their effect on clinicopathological features of breast tumors. Those findings are believed to improve our knowledge on personalized BC prognosis.

Genes *GSTM1, GSTT1*, *GSTP1*, *SULT1A1* and *UGT1A1* encode for enzymes which have the ability to inactivate estrogen metabolites and remove them from the target tissues. The efficiency of estrogen metabolism depends on interindividual genetic variations, polymorphisms, within these genes.

Glutathione S-transferases (GSTs) are a superfamily of enzymes which catalyze the conjugation of glutathione to a variety of chemicals, including estrogen metabolites, making them more water-soluble and easier to excrete. In such a way they decrease oxidative stress generated during estrogen metabolism. There are seven human cytosolic GSTs designated GST Alpha, Mu, Pi, Sigma, Omega, Theta, and Zeta [3]. *GSTM1*, *GSTT1* and *GSTP1* have been the most commonly studied.

*GSTM1* gene includes a deletion polymorphism which frequently affects both alleles. Deletion polymorphism in homozygous state (*GSTM1* null) results in the absence of the enzyme. *GSTT1* gene also has a deletion polymorphism. Homozygotes for the null allele of *GSTT1* lack the respective enzyme. The *GSTP1* gene contains several polymorphisms, including adenine to guanine transition at nucleotide 313 (*A*313*G*) which results in isoleucine to valine substitution in codon 105. The substitution is located in close proximity to the hydrophobic substrate-binding site and was shown to decrease the enzyme’s activity and affinity for electrophilic substrates but not for glutathione [4].

Sulfo-transferases (SULTs) are also involved in inactivation of estrogens by sulfating them to water-soluble metabolites. The most common polymorphism of *SULT1A1* is Arg213His resulting from G to A transition at nucleotide 638. It was shown the His amino acid is associated with lower enzyme activity and lower stability than the wild-type amino acid [5].

UDP-glucuronosyltransferases (UGTs) catalyze the conjugation of glucuronic acid to a variety of compounds including estrogens and their metabolites. One of the most common genetic variant of *UGT1A1* is a dinucleotide repeat in the promoter region of the gene. The wild type allele consists of six TA repeats while the variant allele has seven repeats in the A(TA)nTAA motif. The seven repeat allele is known to be associated with decreased gene expression in comparison to the wild type allele [6]. The (TA)_5_ and (TA)_8_ alleles are rare.

The aim of this study was to examine possible associations between germline genetic polymorphisms in estrogen metabolizing genes and breast cancer clinicopathological features together with disease progression.

## Methods

### Study population

A group of 80 young (≤50 years of age) premenopausal female patients, diagnosed with stage I-II BC was collected. Women, who had additional cancer of any other location, were excluded from the study. Patient enrolment started in 2005 and follow-up was complete until 2012 12 31*.* Blood samples were collected restrospectively during the treatment. The research was approved by Kaunas Regional Biomedical Research Ethics Committee (protocol number BE-2-13) and conducted at the Hospital of *Lithuanian University of Health Sciences, Kaunas Clinics*. Informed consent was obtained from every participant.

Clinicopathological data was collected from medical records for the analysis of differences among genotypes. Tumor estrogen receptor (ER), progesterone receptor (PR) and human epidermal growth factor receptor 2 (HER2) status had been determined immunohistochemically. HER2 was considered positive when imunohistochemically detected HER2 (3+) or (2+) amplification status was confirmed by positive Silver in Situ Hybridization. Tumour receptor status determined the choice of adjuvant therapy for clinicians according to approved prognostic and predictive breast cancer factors and following guidelines for breast cancer treatment at the time.

Most of the studied BC patients (85%) had negative stromal lymphocyte infiltration. Almost half of the cases (48.8%) had positive lymph node involvement. Majority of the tumours (68.8%) were well to moderately differentiated (G1 or G2). Approximately half of the studied BC patients were positive for estrogen (51.2%) and progesterone (46.3%) receptors, while HER2 overexpression was determined only in 22.5% of tumors. The majority of the patients (47.5%) had luminal A tumor subtype. Triple negative, luminal B, HER2 molecular subtypes were observed in 30%, 12.5% and 10% of cases, respectively. Most of the tumours (63.8%) were not larger than 2 cm. During a follow-up period (of at least 2 years) 27.5% of patients were documented with a disease progression. Local and systemic disease spread (excluding contralateral BC) was considered to be disease progression.

### Genotyping

Genomic DNA was isolated from peripheral blood leukocytes using a commercially available DNA extraction kit (ThermoFisher Scientific Baltics, Lithuania) utilizing silica-based membrane technology.

Homozygous deletions of *GSTT1* and *GSTM1* genes were detected by multiplex polymerase chain reaction (PCR). The reaction conditions were described by Altayli and colleagues [7]. Albumin gene (*ALB*) was used as internal positive control. Primer sequences (Table 1) for the studied genes and *ALB* have been previously described by Ambrosone *et al.* [8] and Altayli *et al.* [7], respectively. The absence of *GSTM1* or *GSTT1* amplification indicated null genotypes.

**Table 1:** **Primer sequences and length of PCR/RFLP products**

**Polymorphism**	Primer sequence*	**Length of PCR or RFLP product, bp**	
		**Wild type allele**	**Polymorphic allele**
*GSTM1* null polymorphism	F: 5′-GAACTCCCTGAAAAGCTAAAGC-3′	215	No product
	R: 5′-GTTGGGCTCAAATATACGGTGG-3′		
*GSTT1* null polymorphism	F: 5′-TTCCTTACTGGTCCTCACATCTC-3′	480	No product
	R: 5′-TCACCGGATCATGGCCAGCA-3′		
*ALB (*positive control)	F: 5′-GCCCTCTGCTAACAAGTCCTA′-3	350	-
	R: 5′-GCCCTAAAAAGAAAATCGCCAATC′-3		
*GSTP1* A313G	F: 5′-CCAGTGACTGTGTGTTGATC-3′	189	148; 41
	R: 5′-CAACCCTGGTGCAGATGCTC-3′		
*UGT1A1* A(TA)nTAA	F: 5′-GTCACGTGACACAGTCAAAC-3′	98	100
	R: 5′-TTTGCTCCTGCCAGAGGTT-3′		
*SULT1A1* G638A	F: 5′-GTTGGCTCTGCAGGGTTTCTAGGA-3′	166; 167	333
	R: 5′-CCCAAACCCCCTGCTGGCCAGCACCC-3′		

*F – forward primer, R – reverse primer.

A313G polymorphism in *GSTP1* gene was determined by PCR-RFLP (Restriction Fragment Length Polymorphism) method. DNA fragments were amplified for 37 cycles with annealing at 63°C. Primer sequences (Table 1) have been previously described by Zhao and coauthors [9]. Amplification products were then subjected to *Alw*26I restriction enzyme digestion where presence of the G allele resulted in the generation of 148 and 41 bp fragments, while the A allele remained uncut.

*SULT1A1* G638A polymorphism was investigated using PCR-RFLP. Reaction conditions and primer sequences were designed by Han *et al*. [10]. The digestion with *Hha*I yielded 166 and 167 bp fragments in the presence of wild type allele.

**Figure 1:**
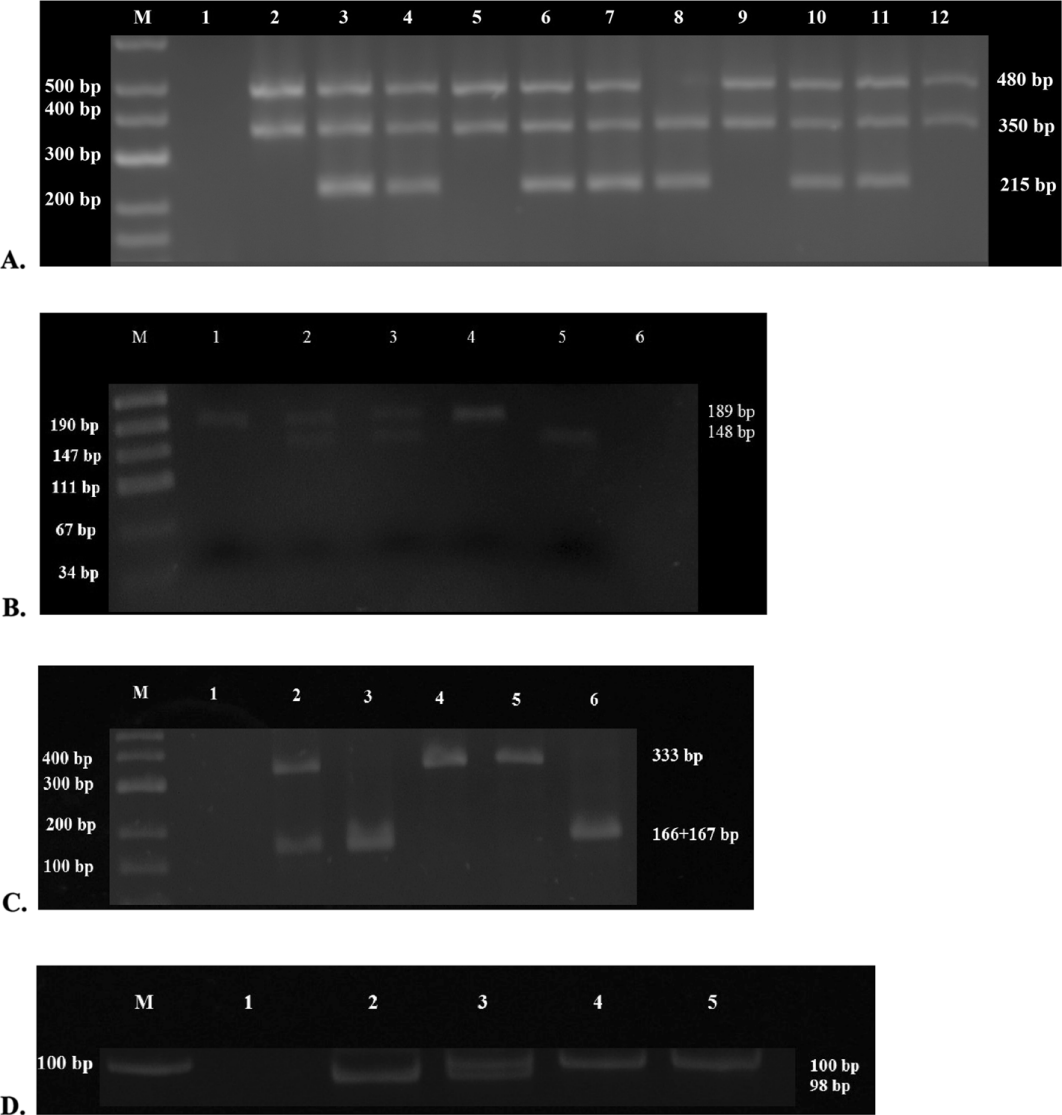
**The electrophoresis results of PCR products and restriction endonuclease digestion. A**. PCR products of *GSTM1* and *GSTT1*. Lane M: DNA Ladder; Lane 1: Negative control (no DNA); Lanes 2, 5, 9, 12: *GSTM1* null, *GSTT1* present; Lanes 3, 4, 6, 7, 10, 11: *GSTM1* present, *GSTT1* present; Lane 8: *GSTT1* null, *GSTM1* present. **B**. Digestion products of *GSTP1*. Lane M: DNA Ladder; Lanes 1 and 4: AA; Lane 2: Heterozygous positive control for digestion; Lane 3: AG; Lane 5: GG; Lane 6: Negative control. **C**. Digestion products of *SULT1A1*. Lane M: DNA Ladder; Lane 1: Negative control; Lane 2: AG; Lane 3: GG; Lanes 4 and 5: AA; Lane 6: Positive control for digestion. **D**. PCR products of *UGT1A1*. Lane M: DNA Ladder; Lane 1: Negative control; Lane 2: 6/6; Lane 3: 6/7; Lanes 4 and 5: 7/7. The DNA ladders are used for approximate sizing of DNA fragments. The discrepancy between the size of digestion products and DNA ladder fragments in panel B may be due to the different fragment migration in gel resulting from different GC/AT content. The 41 bp fragment is extremely faint and masked by the tracking dye in panel B.

All the above PCR products were separated by electrophoresis in 2% agarose gel, stained with ethidium bromide (Figure 1A,B,C).

In order to analyze TA repeats in the promoter of the *UGT1A1* gene PCR reaction was performed with 35 cycles of amplification and with annealing at 56 C. Primer sequences (Table 1) have been previously described by Monaghan *et al*. [11]. PCR products were then subjected to electrophoresis in 12% polyacrilamyde gel. A fragment of 98 bp corresponded to the wild type allele, 100 bp – the seven repeat allele (Figure 1D).

Genotyping results were confirmed by re-genotyping. All the results (100%) of the second genotyping were in agreement with the ones obtained previously.

### Statistical analysis

Prior to association analyses, deviation from Hardy-Weinberg equilibrium (HWE) was tested for each polymorphism using a Chi-square test. Relationships between genotypes and clinicopathological features together with disease progression were estimated by Pearson‘s Chi-square test. In cases where >25% of cells had expected value less than 5, Monte Carlo P values were assessed. Logistic regression analyses were performed to estimate the odds ratios (OR) associating different *GSTM1, GSTT1, GSTP1, SULT1A1* and *UGT1A1* genotypes with clinicopathological characteristics and disease progression. P < 0.05 was considered statistically significant. Association analyses and logistic regression were carried out using SPSS (Statistical Package for the Social Sciences) version 20.0 statistical software (SPSS Inc., Chicago, IL). Statistical power was also calculated when a statistically significant association was found.

## Results

### Genotype distribution of the study population

In a group of 80 patients studied 48.8% were *GSTM1* null and 17.5% were *GSTT1* null. The frequencies of *GSTP1* genotypes were as follows: 52.5% AA, 35.0% AG and 12.5% GG. Regarding *SULT1A1* 43.8% of cases were GG, 46.3% were AG and 10% were AA. *UGT1A1* genotyping identified 43.8% 6/6, 42.5% 6/7 and 13.8% of patients to have 7/7 genotype. The genotypes of *GSTP1*, *SULT1A1* and *UGT1A1* were under HWE. Concerning *GSTM1* and *GSTT1*, the genotyping assay used in this study does not distinquish homozygous wild type from heterozygous individuals, which makes it impossible to evaluate deviation from HWE.

### The associations between genotypes and tumor characteristics

We aimed to determine the linkage between the genotypes of genes involved in estrogen metabolism and clinicopathological characteristics of BC together with disease progression. The results (presented in Additional file 1: Table S2) of the analysis showed a few significant associations.

There was a statistically significant correlation between *GSTT1* genotype and the presence of disease progression (P = 0.038). We also determined a significant association between *SULT1A1* G638A genotype and HER2 molecular subtype of BC (P = 0.016). As far as *GSTM1*, *GSTP1* or *UGT1A1* genes are concerned (Additional file 1: Table S2) we did not identify any significant genotype-phenotype association in our study (by Pearson’s Chi-square test). Synergistic effect of combined *GSTT1* and *GSTM1* genotypes (both null genotypes *versus* other genotypes) was also analyzed, however no statistically significant correlation was determined.

### Dominant and recessive models

Additional analyses were performed to clarify further the effect of low activity variant allele on BC phenotype and disease progression. The dominant and recessive models of each polymorphism are given in Table S3 (see Additional file 1). The results showed *SULT1A1* allele A to be significantly associated with HER2 molecular subtype only in a recessive model (P = 0.006). As our detection method for *GSTM1* and *GSTT1* deletions lack the information on heterozygosity, assessing the models for those polymorphisms was impossible. Other correlations were non-significant.

### The odds ratios

Table 2 presents statistically significant odds ratios determined by logistic regression analysis. The data indicated that individuals with *GSTT1* null genotype have approximately 3.5 times higher risk for BC progression than those with wild type genotype (OR = 3.472, 95% CI 1.043-11.559, P = 0.043). The association remained statistically significant after adjustment for all the treatments the patient received. The prevalence of HER2 BC subtype was significantly higher in individuals with *SULT1A1* AA genotype (OR = 19.971, 95% CI 1.716-232.480, P = 0.017) when compared to GG genotype. A allele significantly increased the chances of HER2 molecular subtype tumours only in a recessive model (OR = 7.996, 95% CI 1.457-43.873, P = 0.017) but not in a dominant one (P = 0.099).

**Table 2:** **Odds ratios for associations of different genotypes with breast cancer clinicopathological features**

**Genotype**	Adjusted* odds ratio (95% Confidence interval)	**P value**
*Odds of disease progress*		
*GSTT1* null genotype *versus* present genotype	3.472 (1.043-11.559)	0.043
*Odds of positive lymph nodes*		
*GSTP1* AG *versus* AA	2.803 (1.049-7.487)	0.040
*Odds of HER2 molecular subtype*		
*SULT1A1* AA *versus* GG	19.971 (1.716-232.480)	0.017
*SULT1A1* AA *versus* AG + GG	7.996 (1.457-43.873)	0.017

*Adjusted for age at diagnosis.

Only statistically significant associations are shown.

In addition, logistic regression analysis showed that *GSTP1* AG genotype significantly increased the odds of positive lymph nodes in comparison to AA genotype (OR = 2.803, 95% CI 1.049-7.487, P = 0.040), although no significant association was detected by Pearson Chi-square test.

In each case of a significant association, statistical power was calculated and varied between 59.5-97.1%.

## Discussion

To our knowledge this is the first study investigating the association between genotypes in five major estrogen metabolizing genes and BC characteristics. We assessed the relationships between *GSTM1*, *GSTT1*, *GSTP1*, *SULT1A1*, *UGT1A1* genotypes and BC phenotype together with disease progression. Our study found significant associations between polymorphisms in BC susceptibility genes and clinicopathological features of BC, as well as disease progression, with corresponding statistical power between 59.5-97.1%. These findings could provide useful prognostic information.

We identified a statistically significant correlation between *GSTT1* null allele and disease progression, which remained statistically significant after adjustment for the treatments the patient received (P = 0.025). However, other associations of *GSTT1* and tumor characteristics were non-significant. Pongtheerat and colleagues [12] also analyzed the linkage between *GSTT1* deletion and lymph node involvement, status of estrogen, progesterone and HER2 receptors, and tumor size. This study was performed in Thai BC patients and no significant association was reported.

Futhermore, our study showed that heterozygous *GSTP1* A313G genotype increases the chances of lymph node involvement if compared with AA genotype. Interestingly, homozygous GG genotype was not significantly related to lymph node status in our study. Bearing in mind the G allele is the low activity allele we anticipated the homozygous variant genotype to associate with positive lymph nodes. The absence of significant association might be due to a small number of individuals with GG genotype in our study. After all, Nedelcheva Kristensen *et al.* [13] found G allele to be associated with negative lymph nodes in a dominant model (AG + GG *versus* AA). However, Pongtheerat *et al.* [12] and Romero *et al.* [14] reported no correlation between *GSTP1* A313G polymorphism and lymph node status in Thai and Spain BC populations, respectively. Prognostic value of axillary node status together with inconsistency between the data about G allele effect on lymph node metastasis points out to a necessity of larger studies in the future.

Regarding *GSTP1* genotype other correlations were non-significant in our study, which is consistent with Romero and colleagues report [14]. However, Pongtheerat and his group [12] identified a relation between *GSTP1* A313G polymorphism and PR status. Since PR expression has a prognotic value and is related to favourable prognosis, further investigation is needed to clarify the effect of *GSTP1* on tumor characteristics.

We revealed that AA genotype of *SULT1A1* increased the odds of HER2 molecular subtype of BC. HER2 amplification/overexpression is a strong prognostic factor for relapse and poor overall survival. HER2 molecular subtype BC is related to poor prognosis of the patients. Our finding that *SULT1A1* AA genotype encoding the low activity enzyme is associated with a poor prognosis factor is in agreement with a general notion that a high sulfation capacity is protective against proliferative effects of estrogens.

Other associations between *SULT1A1* and clinicopathological BC characteristics were not detected in our study. Shatalova *et al.* [15] also did not find any association between *SULT1A1* genotype and BC phenotype, comprising tumour size and lymph node metastasis in BC patients of Russian ancestry. However, Han *et al.* [16] announced that Chinese carriers of A allele had a significantly higher number of positive lymph nodes, thus, arising discussion on topic.

According to our results *GSTM1* null deletion does not play an important role on BC phenotype and disease progression. The results are in agreement with Medeiros *et al*. [17], Romero *et al.* [14] and Lizard-Nacol *et al.* [18] reports, which also did not show the associations regarding *GSTM1* deletion. However, Nedelcheva Kristensen *et al.* [13] reported that combined *GSTM1* null and *GSTT1* null genotype correlated with positive lymph node status. Our study did not determine any association of combined *GSTM1* null and *GSTT1* null genotype with BC phenotype or disease progression.

Furthermore, *UGT1A1* gene did not reveal any significant association with studied tumor characteristics. Shatalova *et al*. [19] also investigated the relationship between *UGT1A1* A(TA)nTAA polymorphism and tumour size and grade but did not find any linkage. However, another study by Shatalova and colleagues [15] reavealed the association between this polymorphism and tumour size in Russian BC cases which encourages futher investigation.

Generally, the observed associations of lack of/ low enzyme activity with poor BC prognosis in our study are in agreement with a perception that lower efficiency of estrogen metabolite elimination leads to a more intense estrogenic stimulation of cancer tissue growth. Nonetheless, our study has some limitations. One of the limitations is small sample size. Larger cohort would allow conducting more precise subgroup analyses by different BC characteristics. Short follow-up period of our patients is another limitation of the study.

## Conclusions

In conclusion, our findings suggest that *GSTT1* null genotype and *SULT1A1* G638A AA genotype could be useful genetic markers for breast cancer prognosis. Further analyses on larger sample size are needed to highlight the effect of *GSTP1* G allele on breast cancer prognosis.

## Additional file

Additional file 1: Table S2. Association between genotype of each polymorphism with clinicopathological features of breast cancer. **Table S3.** Association between variant of each polymorphism (dominant and recessive models) with clinicopathological features of breast cancer.
